# Comparative Proteomic Analysis of *Mycobacterium tuberculosis* Lineage 7 and Lineage 4 Strains Reveals Differentially Abundant Proteins Linked to Slow Growth and Virulence

**DOI:** 10.3389/fmicb.2017.00795

**Published:** 2017-05-09

**Authors:** Solomon A. Yimer, Alemayehu G. Birhanu, Shewit Kalayou, Tahira Riaz, Ephrem D. Zegeye, Getachew T. Beyene, Carol Holm-Hansen, Gunnstein Norheim, Markos Abebe, Abraham Aseffa, Tone Tønjum

**Affiliations:** ^1^Department of Microbiology, Oslo University HospitalOslo, Norway; ^2^Department of Microbiology, University of OsloOslo, Norway; ^3^Department of Medical Biotechnology, Institute of Biotechnology, Addis Ababa UniversityAddis Ababa, Ethiopia; ^4^Centre for Applied Biotechnology, Uni Research EnvironmentBergen, Norway; ^5^Infection Control and Environmental Health, Norwegian Institute of Public HealthOslo, Norway; ^6^Department of Research and Innovation, Armauer Hansen Research InstituteAddis Ababa, Ethiopia

**Keywords:** *Mycobacterium tuberculosis*, tuberculosis, lineage 7, proteomics, Ethiopia, mass spectrometry, type 7 secretion

## Abstract

In order to decipher the nature of the slowly growing *Mycobacterium tuberculosis (M*.tuberculosis) lineage 7, the differentially abundant proteins in strains of *M. tuberculosis* lineage 7 and lineage 4 were defined. Comparative proteomic analysis by mass spectrometry was employed to identify, quantitate and compare the protein profiles of strains from the two *M. tuberculosis* lineages. Label-free peptide quantification of whole cells from *M. tuberculosis* lineage 7 and 4 yielded the identification of 2825 and 2541 proteins, respectively. A combined total of 2867 protein groups covering 71% of the predicted *M. tuberculosis* proteome were identified. The abundance of 125 proteins in *M. tuberculosis* lineage 7 and 4 strains was significantly altered. Notably, the analysis showed that a number of *M. tuberculosis* proteins involved in growth and virulence were less abundant in lineage 7 strains compared to lineage 4. Five ABC transporter proteins, three phosphate binding proteins essential for inorganic phosphate uptake, and six components of the type 7 secretion system ESX-3 involved in iron acquisition were less abundant in *M. tuberculosis* lineage 7. This proteogenomic analysis provided an insight into the lineage 7-specific protein profile which may provide clues to understanding the differential properties of lineage 7 strains in terms of slow growth, survival fitness, and pathogenesis.

## Introduction

Tuberculosis (TB) has claimed an uncountable number of lives over centuries. One third of the global population is infected with the causative agent *Mycobacterium tuberculosis (M. tuberculosis)*, which is the main cause of TB. Each year, ~10.4 million people contract TB and 1.8 million die from the disease (WHO, 2016). In line with the World Health Organization (WHO) vision, the world is united in the quest to eliminate TB by 2050 (WHO, 2016). A holistic approach to understand host, environmental and bacterial factors to TB susceptibility and disease is crucial to achieve the global TB elimination target. There is a large body of evidence on host and environmental attributes for TB (Rieder, 1999). However, given the potential implications for severity of illness and transmission, there is limited knowledge regarding the basic mechanisms underlying the physiology and pathogenesis of the different *M. tuberculosis* lineages.

Large sequence polymorphisms classify *M. tuberculosis* into 7 main lineages. These are lineage 1 (Indo-Oceanic), lineage 2 (East Asian including “Beijing”), lineage 3 (CAS/Delhi), lineage 4 (Euro-American including Latin American Mediterranean (LAM), Haarlem, X type and T families), lineage 5 and lineage 6 (West African 1 and 2, respectively), and lineage 7 (Comas et al., 2013). Lineage 7 was recently detected in Ethiopia and among Ethiopian immigrants in Djibouti (Blouin et al., 2012; Firdessa et al., 2013; Yimer et al., 2013, 2015). We have shown that cells of the *M. tuberculosis* lineage 7 grow slowly *in vitro* and that lineage 7 infections are associated with prolonged delay in seeking health care among patients compared to other lineages (Yimer et al., 2015). Whole genome sequencing (WGS) demonstrated that *M. tuberculosis* lineage 7 cells host a high number of mutations in genes involved in carbohydrate transport and metabolism, transcription, energy production and conversion, all of which contribute to the slow-growth phenotype (Yimer et al., 2016). Former WGS studies have described characteristics and attributes of the other *M. tuberculosis* lineages that may play important roles in the pathogenesis of TB (Coscolla and Gagneux, 2014). For example, the Beijing genotype (lineage 2) is associated with high bacillary load in acid-fast bacillus (AFB) smears and frequently acquires drug resistance (Coscolla and Gagneux, 2014). Lineages 2, 3, and 4 exhibited a lower early inflammatory response compared to lineage 1 and lineage 6 (Chacon-Salinas et al., 2005). Lineage 3 showed a higher anti-inflammatory phenotype compared to Lineage 4 (Portevin et al., 2011). *Mycobacterium africanum* (lineage 6) acquires drug resistance at a lower rate than the Euro-American (lineage 4) in Ghana (Albanna et al., 2011). This shows that even though they are genetically closely related, different strains of *M. tuberculosis* present very diverse clinical phenotypes in terms of virulence. We therefore hypothesized that there may be a mechanism underlying the variation in pathogenicity observed between strains of *M. tuberculosis* detectable at the proteomic level. Global proteomic characterization by use of mass spectrometry represents a powerful tool and is an important supplement to genomics in defining the protein expression patterns (indicating which genes are expressed and down- or upregulated; Peirs et al., 2005; Kelkar et al., 2011; Schubert et al., 2015; Bespyatykh et al., 2016; Jhingan et al., 2016; Peters et al., 2016). To date, no studies have addressed the relationship between the *M. tuberculosis* lineage 7 slow-growth phenotype and its proteomic signatures.

The objective of this study was to characterize the differentially abundant protein profile of *M. tuberculosis* lineage 7 (L7-35 and L7-28) and lineage 4 H37Rv strains and define the proteomic profiles relevant for growth and pathogenicity. This proteomic study generated novel insight into the differentially abundant (DA) proteins in *M. tuberculosis* lineage 7 vs. lineage 4 strains. A total of 2,867 proteins covering 71% of the predicted *M. tuberculosis* proteome were identified. The analysis of DA proteins by pathway clustering provided an overview on the lineage-specific protein interaction profile. This information may be used to explain the differential behavior of lineage 7 strains in terms of slow growth, survival fitness, and pathogenesis.

## Materials and methods

### Mycobacterial strains and growth conditions

Whole genome sequenced *M. tuberculosis* lineage 7 strains (L7-35 and L7-28) collected form the Amhara Region of Ethiopia and lineage 4 strain H37Rv were streaked onto Middlebrook 7H10 plates in triplicates from freezer stocks, and incubated in a humidified 37°C, 5% CO_2_ incubator. After 32 days the cells were carefully scrapped off the agar plates and put into 50 mL Falcon® tubes. The cell pellets were gently resuspended in 30 mL PBS, pH 7.4, and centrifuged at 3,900 rpm for 20 min at 4°C. The cell pellets were subsequently transferred into 2 mL screw capped tubes (Sarstedt, Nümbrecht, Germany) and resuspended in 1 mL PBS, and heat inactivated at 80°C for 90 min. Culturing and processing of the *M. tuberculosis* samples up until the heat inactivation step were conducted in a biosafety level 3 facility at Oslo University Hospital, Norway. The heat-inactivated *M. tuberculosis* samples were stored at −80°C until lysed for mass spectrometry analysis.

### Proteomic analysis

#### Cell lysis

The heat-inactivated cell pellets were resuspended in lysis buffer containing 2% SDS, 10 mM Tris-HCl (pH 7.5), 1 tablet per 50 mL EDTA-free Protease Inhibitor Cocktail (Sigma-Aldrich, Cleveland, US) and 1 tablet per 10 mL PhosSTOP Phosphatase Inhibitor Cocktail (Roche). The samples were subsequently transferred into Lysing Matrix B tubes (Roche) and disrupted mechanically by bead beating using MagNa Lyser (Roche Diognostics, GmbH, Mannheim, Germany) for 90 s, speed 6.0. The lysis procedure followed by 1 min cooling on ice was repeated six times. The lysate was clarified by centrifugation (15,000 × g for 15 min) at 21°C, and the supernatant containing the whole cell lysate proteins was transferred in to new 2 mL screw cap micro tubes (Sarstedt, Nümbrecht, Germany).

#### In-gel trypsin digestion of cellular proteins

Hundred μg of protein sample dissolved in NuPAGE LDS sample buffer (4x) and NuPAGE Sample Reducing Agent (10X) (Life Technologies, USA) were incubated for 10 min at 70°C and pre-fractionated by 1.0 mm, 4–12% NuPAGE Novex Bis-Tris SDS-PAGE gel (Life Technologies), at 80 V for 5 min followed by 20 min at 200 V. Gels were Coomassie-stained using a Colloidal Blue Staining kit for NuPAGE as per manufacturer's instructions. After staining, each gel lane was divided into 6 fractions, and each fraction was subjected to in-gel reduction, alkylation, and tryptic digestion (Shevchenko et al., 2006). In brief, proteins were reduced using 10 mM DTT for 1 h at 56°C and alkylated with 55 mM iodoacetamide for 1 h at room temperature (Sigma-Aldrich, Cleveland, US). The reduced and alkylated peptides were digested with sequencing-grade trypsin (Promega, WI, USA, 1:100; w/w) for 16 h at 37°C in 50 mM NH_4_HCO_3_. The trypsin-digested protein samples were extracted from the gel using sequential (50 and 100%) acetonitrile (ACN), dried by SpeedVac concentrator (Eppendorf, concentrator 5301) and re-suspended using 0.05% trifluoroaceticacid (TFA). For desalting, the peptide samples were loaded on to C_18_ stage tips activated and equilibrated with 95% ACN/0.1% FA and 0.1% formic acid (FA), respectively. The loaded samples were washed with 0.05% TFA and eluted with 95% ACN/0.1% FA. The eluent was dried using a SpeedVac concentrator, re-suspended in 0.1% FA, transferred to auto-sampler nano-liquid chromatography (LC) vials and stored at −20°C. The proteomic work flow is depicted in Supplementary file 1.

#### Mass spectrometry (MS) analysis

Peptide identification and quantitation were performed by label-free quantification (LFQ) LC-MS/MS using a Q Exactive hybrid quadropole-orbitrap instrument interfaced with an EASY 1000-nano-LC electrospray ion source (Thermo-Fisher Scientific, Biberach, Germany). Peptides were injected in triplicates into a pre-analytic column (Acclaim PepMap 100, 75 μm × 2 cm, nanoviper, C18, 3 μm, 100 Å, Thermo Fisher Scientific) and separated on an analytical column (PepMap RSLC, C18, 2 μm, 100 Å, 50 μm × 15 cm, Thermo Fisher Scientific) with a 75 min solvent gradient and flow rate of 0.3 μL/min at 60°C. The gradient used was from 2 to 30% solvent B for 30 min followed by 30–75% solvent B from 30 to 35 min and 75 to 90% solvent B from 35 to 70 min. Thereafter the gradient was kept at 90% solvent B from 70 to 75 min, using 0.1% formic acid (FA) in 3% acetonitrile (ACN) as solvent A and 0.1% FA in 97% ACN as solvent B (FA: LC-MS grade, Fluka; ACN: LC-MS grade, Merck Laboratories). The MS instrument was operated in the data-dependent acquisition mode with automatic switching between MS and MS/MS scans. The full MS scans were acquired at 70 K resolution, with automatic gain control target of 1 × 10^6^ ions, maximum injection time of 200 ms and MS scan range 300–1,800 m/z. Higher energy collision dissociation (HCD) was used for peptide fragmentation with normalized collision energy set to 28. The MS/MS scans were performed using a data-dependent top10 method at a resolution of 17.5 K with an automatic gain control target of 5 × 10^4^ ions at maximum injection time of 100 ms and isolation window of 2.0 m/z units. An under fill ratio of 10% and dynamic exclusion duration of 30 s were applied. The mass spectrometry proteomics data have been deposited to the ProteomeXchange Consortium (http://proteomecentral.proteomexchange.org) via the PRIDE partner repository [1] with the dataset identifier PXD006117.

#### Database search

The MS/MS data analysis was performed using the MaxQuant (MQ) software package (version 1.4.0.5; Cox and Matthias, 2008) for analyzing large MS/MS data sets, employing its integrated Andromeda search algorithms (Cox et al., 2011). The raw spectral data were searched against the *M. tuberculosis* H37Rv reference proteome UP000001584 (UniProt-proteome) using reverse decoy databases and a selection of known contaminants provided by MQ. The following parameters were applied for the database search: Enzyme specificity was set as Trypsin/P, and a maximum of two missed cleavages and a mass tolerance of 0.5 Da for fragment ion were applied. The “re-quantify” and “match between runs” options were utilized with a retention time alignment window of 3 min. Carbamidomethylation of cysteine was set as a fixed modification and acetylation of the protein N-terminus, conversion of N-terminal glutamine and glutamic acid to pyroglutamic acid and oxidation of methionine were set as variable modifications for database searches. The first search for precursor ions was performed with a mass tolerance of 20 ppm for calibration, while 6 ppm was applied for the main search. For protein identification, at least 1 unique peptide was required per protein group (Cox and Matthias, 2008; Zhao and Lin, 2010). Minimum peptide length of 7 amino acids was required for identification. The maximum false discovery rate (FDR) cutoff of 0.01 (1%) was set at both the peptide spectra matches and the protein group levels. For all other parameters, the default setting was applied. Following protein identification by a database search, validation for multiple comparisons was corrected using Benjamini-Hochberg correction (Benjamini et al., 2001). To aid in the control of false positives, the database was supplemented with additional sequences for common contaminants and reversed sequences of each entry.

#### Bioinformatics analysis

Bioinformatics analysis was performed using the Perseus software (version 1.5.1.6) as previously described (Tyanova et al., 2016). The protein groups output from MQ was used as the basis for all the subsequent statistical and ontology enrichment analysis. LFQ intensities were used to assess differences in the abundance of proteins between the two *M. tuberculosis* lineages. Abundance estimation of the proteins identified was performed using intensity-based absolute quantification (iBAQ) values. Briefly, the protein groups output was filtered by removing matches to the reverse database, matches only identified by site, and common contaminants. Subsequently, LFQ intensities were transformed to log_2_. For quantitative comparisons, three technical replicates of each biological experiment (*n* = 3) were averaged based on their median values. An LFQ intensity category was then created that consisted of the intensities from two clinical isolates (*M. tuberculosis* L728 and L735) of lineage 7. This merger served as a core proteome for lineage 7, which was later used to compare against the H37Rv lineage 4 reference strain. For statistical analysis, at least two valid LFQ intensities out of the three biological experiments were required. Signals that originally were zero (missing values) were imputed with random numbers from a normal distribution. The mean and standard deviation were chosen to best simulate low abundance values below the noise level (width = 0.3; shift = 1.8; Hubner et al., 2010). A two-tailed unpaired *t*-test with an FDR value of 0.05 and S_0_ = 2 was applied to identify proteins for which the abundance was significantly changed between the two *M. tuberculosis* lineages (Tusher et al., 2001). The resulting *t*-test -significant proteins for each *M. tuberculosis* lineage were analyzed for annotation enrichments. A two-tailed Fisher's Exact Test was used to assess the significance of enrichment terms. Proteins assigned to enriched term categories (*p* < 0.05) were grouped according to the Kyoto Encyclopedia of Genes and Genomes (KEGG) classification. The GO and KEGG categories of proteins identified were added using the Uniprot annotation for the *M. tuberculosis* reference proteome database (Gene ontology^1^).

#### Protein interaction and network analysis

The Search Tool for the Retrieval of Interacting Genes version 10.0 (Franceschini et al., 2013; STRING, http://string-db.org/) was used to interpret the biological significance of DA proteins in terms of predicted protein-protein interaction networks. The required minimum interaction score of at least 0.4 was used as the cut-off point criterion. The Cytoscape software (Shannon et al., 2003) was used to visualize the interaction network predicted. A list of DA proteins, the official gene identifier, and the corresponding relative abundance value were separately uploaded to Cytoscape (http://www.cytoscape.org/). Properties of the network including node degree and edge attributes were then analyzed. Nodes represent proteins and edges represent the interactions/connections between the proteins. The degree represents the number of interactions associated with the protein. Proteins with a large degree are known as hub proteins (Azuaje et al., 2010) and are considered to be the essential or key proteins in the network (Ideker and Sharan, 2008). The Network Analyzer option in Cytoscape 3.4.1 was used to compute the degree and *between-ness* centrality of the network (Assenov et al., 2008). The MCODE program was used to identify the most inter-connected nodes (Bader and Hogue, 2003).

### Ethics approval

The study obtained ethics approval from the Regional Committee for Medical Research Ethics in Eastern Norway (REK Øst) and the Ethiopian Science and Technology Ministry in Addis Ababa, Ethiopia. Written informed consent was obtained from the study participants before the study was conducted.

## Results

### Comprehensive proteome analysis of *M. tuberculosis* lineage 7 and lineage 4 strains

**Figure 1:**
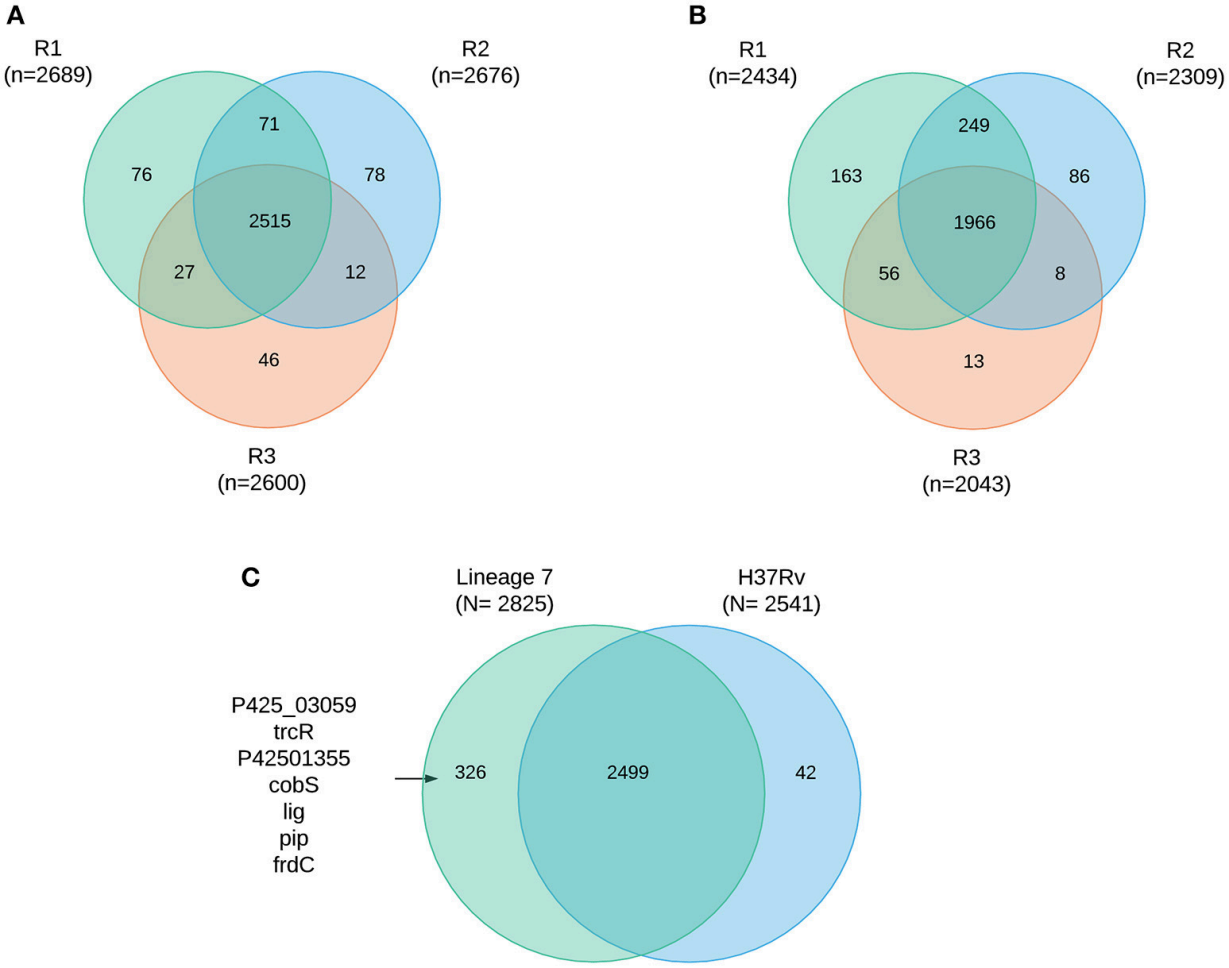
**Protein coverage Venn plot illustrating protein identification overlaps among the three biological replicates (R1–R3) in *M. tuberculosis* lineage 7 (A)** and in lineage 4 **(B)** isolates, **(C)** overlap of protein identification between lineages and some of the mutually exclusively identified proteins.

A total of 2867 *M. tuberculosis* proteins were identified with 99% confidence at the peptide and protein levels (Supplemental file 2) representing 71% protein coverage of the predicted *M. tuberculosis* proteome. The total number of proteins identified in *M. tuberculosis* lineage 7 (L7-35 and L7-28) and in lineage 4 were 2,825 and 2,541, respectively. Among the 2,825 identified proteins in lineage 7 strains, 2499 (87%) proteins were shared in all the biological experiments (Figures 1A–C). Mutual exclusivity analysis revealed 326 and 42 strain-specific protein groups in lineage 7 and lineage 4, respectively (Supplemental file 2), and the main DA component pathways are addressed below. The overlap in protein identification in the different biological replicates and *M. tuberculosis* lineages is shown in the Venn diagram (Figures 1A–C). Of the annotated components, 1,783 (62%) have an assigned molecular function, 1,110 (38.7%) are involved in known biological processes, 948 (33%) are assigned by cellular compartment, and 829 (28.9%) have an assigned KEGG function. The complete list of protein groups is presented in Supplemental file 3.

### Protein profile of *M. tuberculosis* lineage 7 (L7-35 and L7-28) and lineage 4 strains

**Figure 2:**
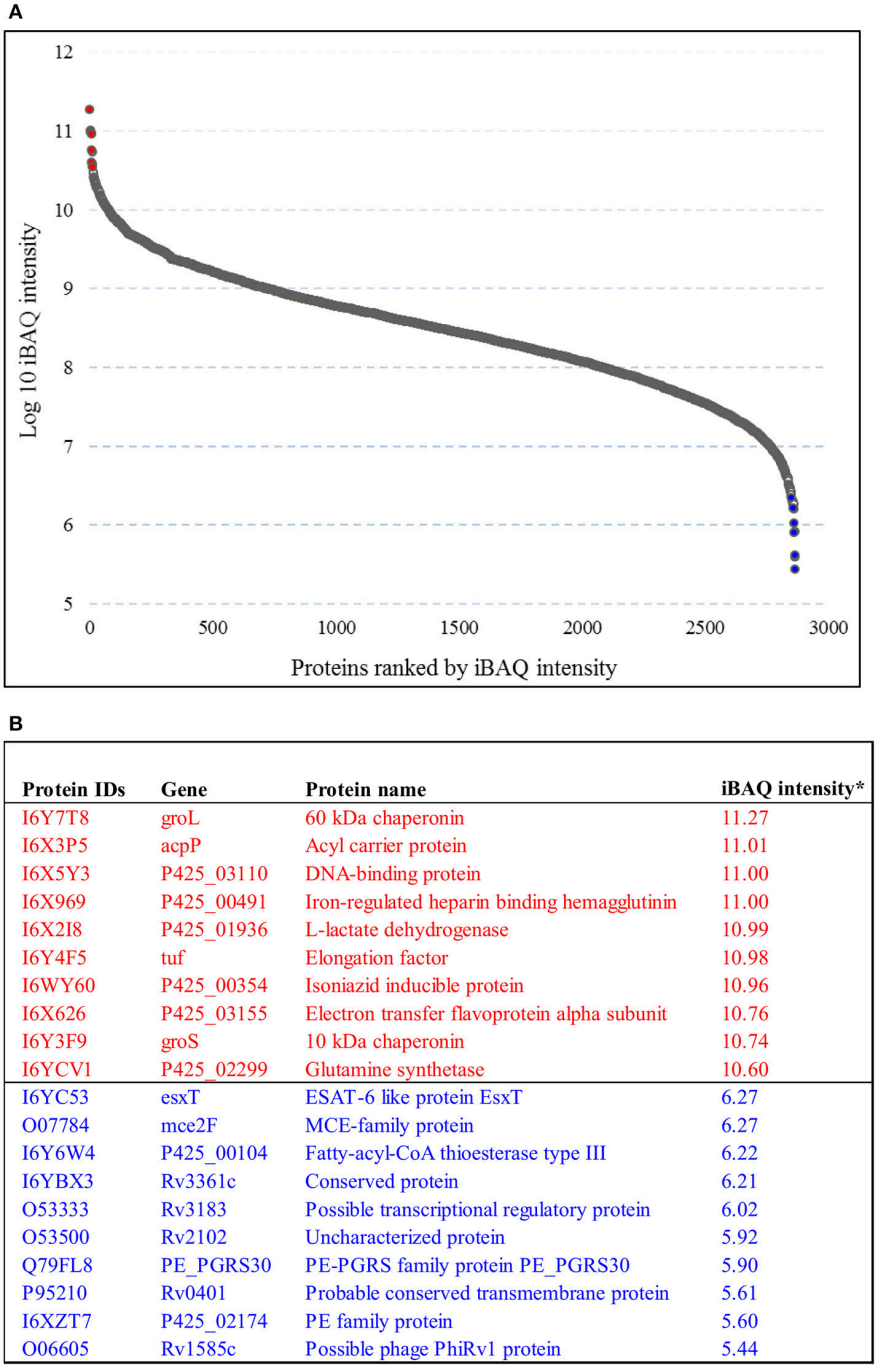
**Protein dynamic range estimation**. **(A)** Combined intensity based absolute quantification (iBAQ) values for the 2,825 proteins were plotted with log_10_ iBAQ intensity on the y axis, and proteins were ranked by iBAQ intensity on the x axis. The plot shows a dynamic range of 6 orders of magnitude. **(B)** List of the ten most (colored red) and 10 least (colored blue) abundant proteins based on iBAQ intensity.

The abundance of the *M. tuberculosis* proteins identified was quantified by iBAQ. This technique takes into account the normalization and summation of MS/MS signals in relation to peptide size, length, and number of theoretical peptides considered acceptable for all the proteins that are defined in a specific proteome run. The 10 most abundant protein classes identified in lineage 7 strain were chaperones, hydrolase isomerase, ligase, oxidoreductase, transfer carrier protein, and nucleic acid-binding proteins. The least abundant proteins identified include the ESAT-6-like protein (EsxT), MCE-family protein (Mce2F), PE-PGRS family protein and PE family protein. The iBAQ intensity in the aggregate proteome covered a dynamic range of six orders of magnitude between the most abundant and least abundant proteins (Figures 2A,B; Supplemental file 3).

**Figure 3:**
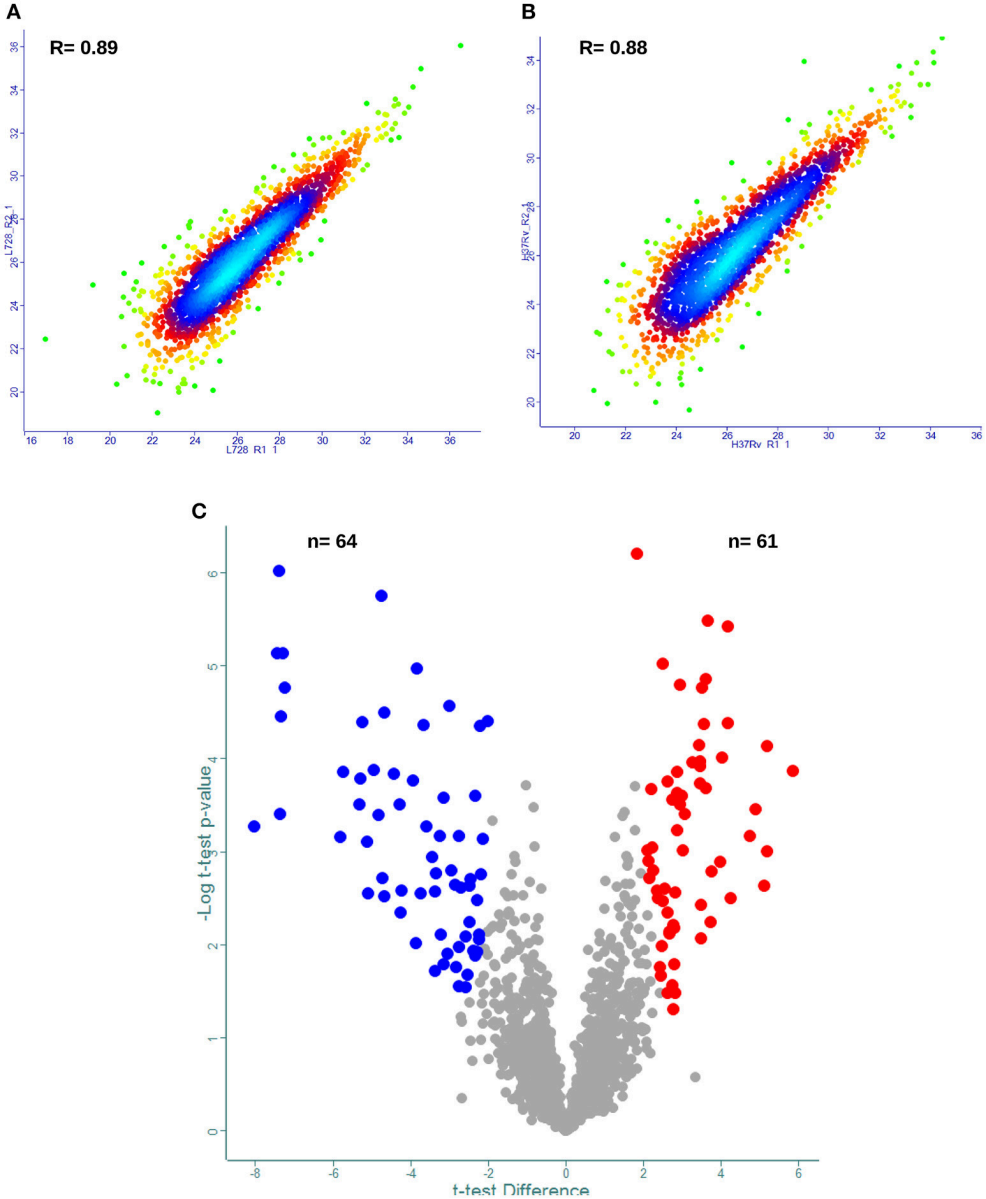
**Label free proteome quantification of *Mycobacterium tuberculosis* lineages**. Quantitative analysis was performed using the MaxQuant and Perseus software environments as described in the method section. Reproducibility of the analytic workflow for biological replicates was assessed by Pearson correlation coefficients (*R*-values). **(A,B)** Represent density scatter plot of peptide intensities for biological replicates of *M. tuberculosis* lineage 7 and lineage 4, respectively. **(C)** volcano plot of protein abundance differences as a function of statistical significance (*t*-test *p* ≤ 0.05 and fold change cutoff point ±2) between lineage 7 and lineage 4 isolates. Y-axis indicates *p*-value (−log_10_). X-axis shows protein ratio (x-axis) in lineage 7 vs. lineage 4 stains. The color code indicates upregulation (red) and downregulation (blue). Proteins with no statistically significant difference in abundances between the two lineages are shown in gray.

After identifying the 2,867 proteins in the composite *M. tuberculosis* proteome, the reproducibility of our label-free quantification workflow was assessed (Supplemental file 1). The Pearson correlations (*R*-values) of biological replicates using normalized protein LFQ intensities were computed. The analysis showed that the *R*-value between normalized intensities was high (Figures 3A,B, Supplemental file 4) and thus was suitable for accurate comparisons of protein abundance differences.

For DA protein comparisons, criteria were set that fulfill two valid LFQ intensity values from each biological triplicate. This resulted in a total of 1,946 proteins. Using a set of statistical criteria, *T*-test *p*-value 0.05, S_0_ = 2 and fold change cutoff point ±2, we found the abundances of 125 proteins to be significantly changed (Figure 3C, Supplemental file 5).

### The proteomes of the *M. tuberculosis* lineage 7 strains are significantly different from lineage 4

**Figure 4:**
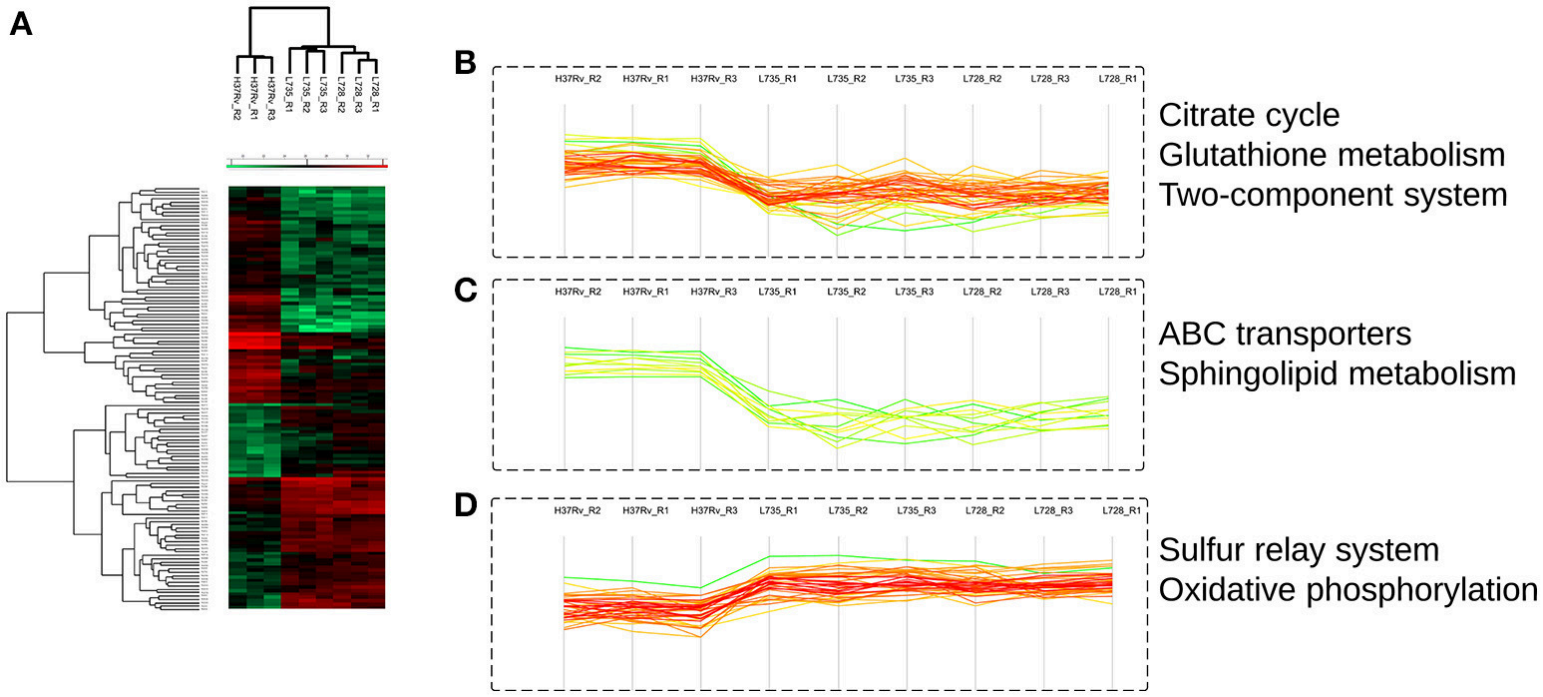
**Quantitative proteomics analysis reveals distinct pattern of *M. tuberculosis* differentially abundant proteins that are involved in several important pathways. (A)** Unsupervised hierarchical clustering representing the *T*-test significant proteins (*n* = 125). Color code indicates the normalized median abundance of the proteins belonging to the category (red more abundant; green less abundant). **(B–D)** Representative clusters of significantly enriched KEGG pathways.

**Figure 5:**
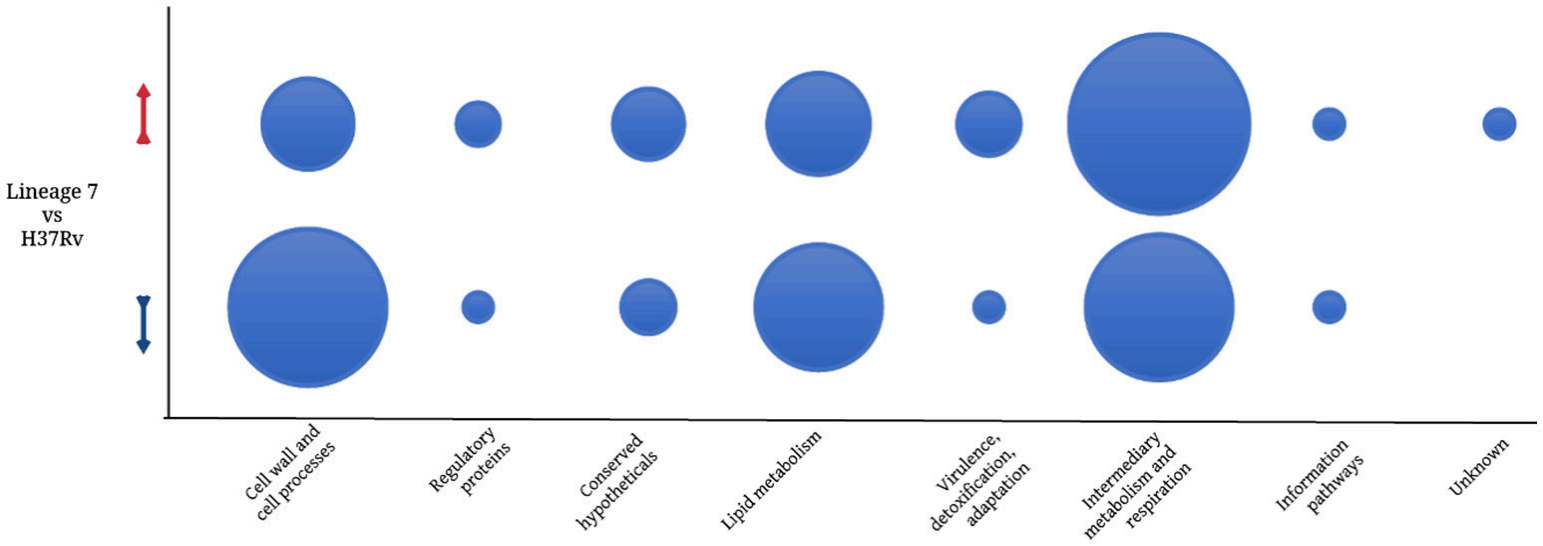
**Bubble plot comparing the number of *M. tuberculosis* differentially abundant proteins in each functional category**. Red arrow denotes upregulation and the blue arrow indicates downregulation. Functional categorization was performed according to TubercuList v 2.6 (http://tuberculist.epfl.ch/).

Among the 125 differentially regulated proteins, 64 were downregulated and 61 proteins were upregulated (Figure 3C, Supplemental file 5). The 125 DA protein groups were further subjected to unsupervised hierarchical cluster analysis. The resulting cluster-gram is shown in Figure 4A. KEGG analysis was then conducted to investigate whether these proteins were enriched for any particular pathway. The pathway sub-clusters that were significantly enriched are shown in Figures 4B–D (Supplementary file 6). The DA proteins were also categorized into their functional categories as defined by TubercuList. A majority of the proteins detected belong to categories of intermediary metabolism and respiration (39.2%), lipid metabolism (20%), cell wall- and cell processes-related (24.8%), conserved hypotheticals (2.4%) and unknowns (0.8%) (Supplemental file 7). As shown in Figure 5, the number of less abundant proteins involved in cell wall and cell processes is greater in lineage 7 strains than in lineage 4. In addition, the number of more abundant proteins involved in intermediary metabolism and respiration is greater in lineage 7 strains than in lineage 4.

**Figure 6:**
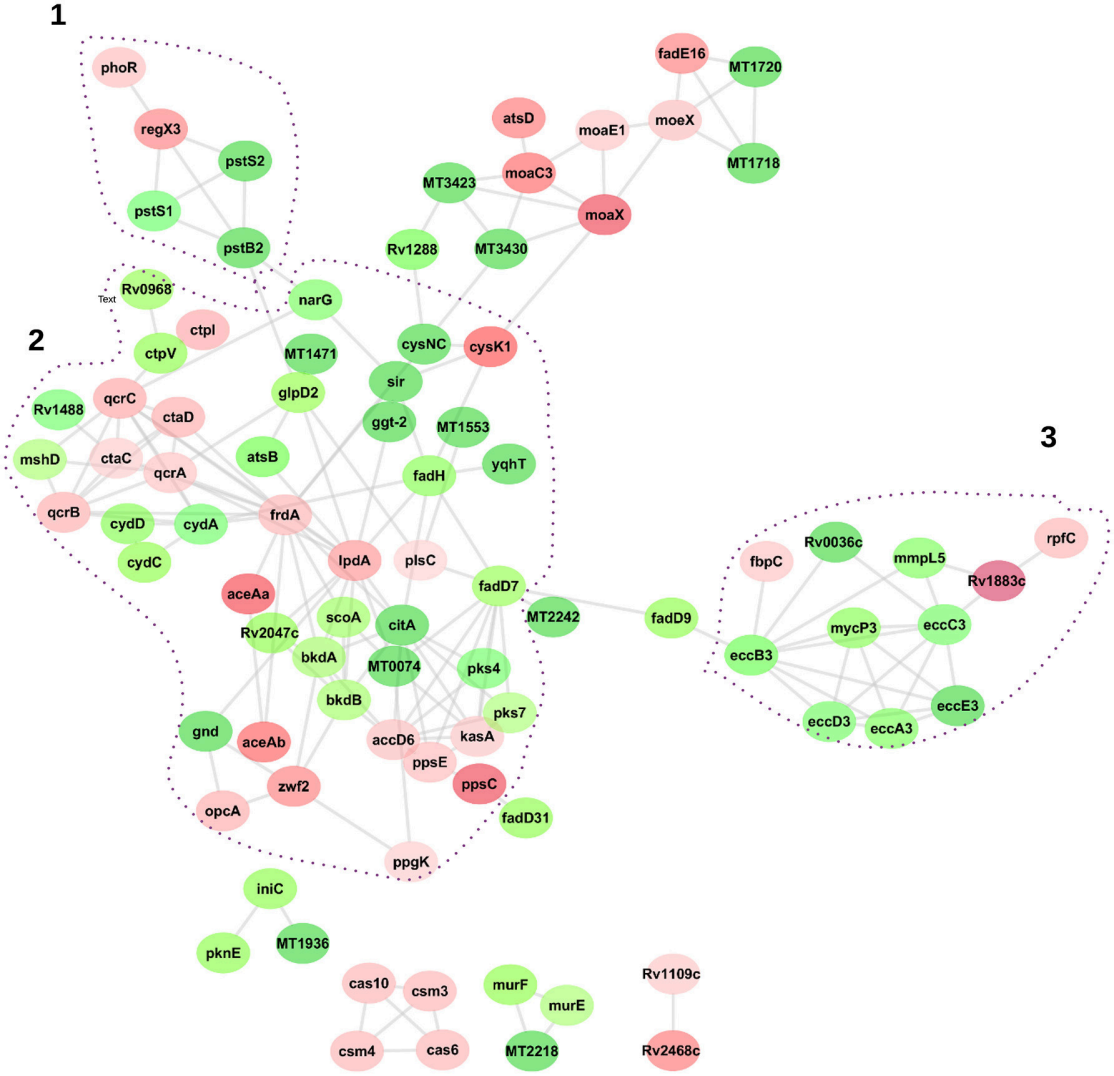
**Network interaction analysis of differentially abundant proteins related to *M. tuberculosis* lineages 7 and 4**. Graphical representation of the interaction was generated by the Cytoscape software version 3.4. Proteins are represented as nodes, and the biological relationship between two nodes is represented as an edge (line). The intensity of the node color indicates the increased (red) or decreased (green) abundance according to fold changes. Dot-lined circles indicate sub-networks of proteins linked to specific KEGG pathways.

To visualize the functional and molecular interaction network, a total of 125 DA proteins were searched into the online STRING protein query database. Eighty eight of the 125 proteins were present in recognized and predicted networks with total interaction edges of 174 interaction networks. Figure 6 suggests that LpdA, FrdA, EccB3, EccC3, and PstB2 represent a significant protein hub. Three network sub clusters of proteins were highly correlated to functions of Pst system, Esx-3 secretion machinery, energy metabolism, and oxidative stress responses (Figure 6).

Cluster 1 in Figure 6 shows interactions of ABC transporter proteins Rv0932c (Pst1), (PstS2), Rv0933 (PstB), and Rv0292 which are essential for the high-affinity capture of periplasmic inorganic phosphate (Pi) in *M. tuberculosis*. These proteins were significantly downregulated with a fold change ranging from 31 to 262. In addition, Rv0491 (RegX3) and Rv0758 (PhoR) part of the two-component regulatory system were upregulated (Table 1). The Lineage 7 strains also exhibited downregulation of Rv3881c (EspB), Rv1196 (PPE18), Rv1743 (PknE), and Rv1980c (Mpt64), which play important roles in the ESX-1 secretion system and apoptosis (Table 2).

**Table 1:** **List of the differentially abundant proteins involved in the Pst system in *M. tuberculosis* lineage 7 (L7-35 and L7-28) vs. lineage 4 (H37Rv) strains**.

**Protein name**	**Protein IDs**	**Gene name**	**Rv number**	**Fold change**
Phosphate-binding protein PstS	I6Y569	pstS2	Rv0932c	−261.84
Phosphate import ATP-binding protein PstB	I6XWL3	pstB	Rv0933	34.46
Phosphate-binding protein PstS	I6XA55	pstS1	Rv0934	−31.02
Two-component system sensory transduction protein	I6Y7Y4	regX3	Rv0491	12.09
Two component system response sensor kinase PhoR	P71815	phoR	Rv0758	5.9

**Table 2:** **List of the *M. tuberculosis* differentially abundant PPE proteins in *M. tuberculosis* lineage 7 (L7-35 and L7-28) vs. lineage 4 (H37Rv) strains**.

**Protein name**	**Protein IDs**	**Gene symbol**	**Rv number**	**Fold change**
PPE family protein PPE18	L7N675	PPE18	Rv1196	−34.87
Secreted ESX-1 substrate protein B EspB	I6YHD6	espB	Rv3881c	−8.29
Immunogenic protein MPT64	I6YC43	mpt64	Rv1980c	−10.35
PPE family protein PPE51	I6YBA9	PPE51	Rv3136	−10.15
Conserved protein	O06216	Rv2161c	Rv2161c	−156.57

Cluster 3 in Figure 6 depicts interactions of the type 7 secretion system (Esx-3) components that are crucial for iron acquisition in *M. tuberculosis*. The ESX-3 secretion machinery proteins (Rv2083 Rv0282 (EccA3), (EccB3), Rv0284 (EccC3), (Rv0290/EccD3), and Rv0292 (EccE3) were downregulated with a fold change ranging from 19 to 55. In addition, Rv0291 (MycP3), Rv0676c (MmpL5), and Rv0036 proteins were downregulated in lineage 7 strains. Rv1884c (RpfC), a resuscitation-promoting factor protein, was upregulated in lineage 7 strains compared to lineage 4 (Table 3).

**Table 3:** **List of the *M. tuberculosis* differentially abundant proteins and corresponding fold changes involved in type 7 secretion system**.

**Protein name**	**Protein IDs**	**Gene symbol**	**Rv number**	**Fold change**
ESX-3 secretion system protein EccA3	I6Y3E7	eccA3	Rv0282	−19.12
ESX-3 secretion system protein EccC3	I6X8X9	eccC3	Rv0284	−25.51
ESX-3 secretion system protein EccD3	I6Y7G0	eccD3	Rv0290	−25.68
ESX-3 secretion system protein EccB3	I6XUX6	eccB3	Rv0283	−37.78
ESX-3 secretion system protein EccE3	I6Y3F4	eccE3	Rv0292	−55.98
Uncharacterized protein	I6X8C7	Rv0036c	Rv0036c	−161.42
Membrane-anchored mycosin MycP	O53695	mycp3	Rv0291	−13.40
Fatty-acid–CoA ligase FadD9	Q50631	fadH9	Rv2590	−7.14
Resuscitation-promoting factor RpfC	I6XZ79	rpfC	Rv1884c	6.82
Diacylglycerol acyltransferase	I6Y2U9	fbpC	Rv0129c	5.47
Transmembrane transport protein MmpL5	I6XVY5	mmpl5	Rv0676c	−19.5
Uncharacterized protein	O07748	Rv1883c	Rv1883c	57.35

Several proteins, Rv2952 (AhpD), Rv0969 (CtpV), Rv0968 (MshD), Rv1161 (NarG), Rv2394 (GgtB), CysD, CysN, CysH, and SirA that are involved in counteracting the effect of reactive oxygen intermediates (ROI) and/or reactive nitrogen intermediates (RNI) were less abundant in lineage 7 (L7-35 and L7-28) compared to lineage 4. Lineage 7 strains also showed downregulation of ABC transporter proteins Rv1620c (CydC) and Rv1621c (CydD). In contrast, a number of proteins that counteract the detrimental effects of ROI and RNI were upregulated in lineage 7 (L7-35 and L7-28) isolates compared to lineage 4. These included GnD1, ZwF1, Rv3119 (MoaE1), RV3324C (MoaC), RV3323C, and (MoaX) proteins (Table 4).

**Table 4:** **List of the differentially abundant proteins involved in ROI and RNI stress exposure responses in *M. tuberculosis* lineage 7 (L7-35 and L7-28) vs. lineage 4 (H37Rv) strains**.

**Protein name**	**Protein IDs**	**Gene symbol**	**Rv number**	**Fold change**
Uncharacterized protein	O07748	Rv1883c	Rv1883c	57.35
MoaD-MoaE fusion protein MoaX	Q6MWY3	moaX	Rv3323c	36.35
Cysteine synthase	I6Y910	cysK1	Rv2334	18.13
Isocitrate lyase AceAb	O07717	aceAb	Rv1916	16.33
Cyclic pyranopterin monophosphate synthase accessory protein	I6YBT7	moaC3	Rv3324c	15.71
Glucose-6-phosphate 1-dehydrogenase	I6XBH9	zwf2	Rv1447c	12.15
Arylsulfatase AtsD	I6XVW9	atsD	Rv0663	13.45
Glucose-6-phosphate 1-dehydrogenase	I6XBH9	zwf2	Rv1447c	12.15
Fumarate reductase FrdA	I6YAW6	frdA	Rv1552	6.83
Molybdenum cofactor biosynthesis protein E1 MoaE1	I6X6B1	moaE1	Rv3119	5.36
Probable component linked with the assembly of cytochrome transport transmembrane ATP-binding protein ABC transporter CydC	O06137	cydC	Rv1620c	−6.00
Respiratory nitrate reductase alpha chain NarG	I6Y9T4	narG	Rv1161	−21.51
Arylsulfatase AtsB	O65931	atsB	Rv3299c	−39.47
Conserved protein	O06216	Rv2161c	Rv2161c	−156.57
Methyltransferase	I6XFR4	Rv2952	Rv2952	−164.28

The DA proteins CitA, BkdB BkdA, FrdA, and LpdA play important roles in the citrate cycle (TCA) pathway were differentially regulated. While CitA, BkdB, BkdA proteins were less abundant, the FrdA and Rv3303c (LpdA) proteins were upregulated in lineage 7 strains compared to lineage 4. Rv3043c (CtaD), Rv2200c (CtaC), QcrA (Rv1446c), QcrC (Rv2194), QcrB (Rv2196), which are the major respiratory route in mycobacteria, were also upregulated. In addition, the Rv1915 (AceaA) and Rv1916 (AceaB) GnD1 and PpgK proteins were upregulated in lineage 7 strains (Table 5).

**Table 5:** **List of the differentially abundant proteins involved in energy metabolism in *M. tuberculosis* lineage 7 (L7-35 and L7-28) vs. lineage 4 (H37Rv) strains**.

**Protein name**	**Protein IDs**	**Gene symbol**	**Rv number**	**Fold change**
6-phosphogluconate dehydrogenase, decarboxylating	Q79FJ2	gnd1	Rv1844c	17.85
Isocitrate lyase AceAa	O07718	aceAa	Rv1915	29.61
NAD(P)H quinone reductase LpdA	I6XGU5	lpdA	Rv3303C	9.52
Isocitrate lyase AceAb	O07717	aceAb	Rv1916	16.33
Arylsulfatase AtsD	I6XVW9	atsD	Rv0663	13.45
Ubiquinol-cytochrome C reductase QcrC	I6Y059	qcrC	Rv2194	8.09
Cytochrome C oxidase polypeptide I CtaD	I6YAZ7	ctaD	Rv3043c	7.61
Ubiquinol-cytochrome C reductase QcrB	I6YCT0	qcrB	Rv2196	7.25
Fumarate reductase FrdA	I6YAW6	frdA	Rv1552	6.83
Rieske iron-sulfur protein QcrA	I6XDR2	qcrA	Rv2195	5.62
Transmembrane cytochrome C oxidase subunit II CtaC	I6XDR7	ctaC	Rv2200c	4.57
Polyphosphate glucokinase PpgK	I6YE62	ppgK	Rv2702	4.42
Gamma-glutamyl transpeptidase GgtB	P71750	ggtB	Rv2394	−4.76
Branched-chain keto acid dehydrogenase E1 component alpha subunit BkdA	I6YDK3	bkdA	Rv2497c	−4.93
Glycerol-3-phosphate dehydrogenase	I6Y352	glpD2	Rv3302c	−5.84
Branched-chain keto acid dehydrogenase E1 component beta subunit BkdB	I6XEG1	bkdB	Rv2496c	−5.02
Citrate synthase II CitA	I6Y908	citA	Rv0889c	−150.96

Several proteins involved in cell wall/lipid biosynthesis were less abundant in lineage 7 than in lineage 4. The RV2952 was markedly downregulated with a 164-fold change in lineage 7 strains. Furthermore, the MmaA3, IniC, Pks4, MurE, and MurF proteins were less abundant in lineage 7 strains. The PpsE PpsC, AccD6, and KasA proteins were upregulated in lineage 7 than in lineage 4 (Table 6).

**Table 6:** **List of the differentially abundant proteins involved in cell wall/lipid biosynthesis in *M. tuberculosis* lineage 7 (L7-35 and L7-28) vs. lineage 4 (H37Rv) strains**.

**Protein name**	**Protein IDs**	**Gene symbol**	**Rv number**	**Fold change**
UDP-N-acetylmuramoyl-tripeptide–D-alanyl-D-alanine ligase	I6YCL0	murF	Rv2157c	−6.01
UDP-N-acetylmuramoyl-L-alanyl-D-glutamate–2,6-diaminopimelate ligase	I6X3E1	murE	Rv2158c	−4.64
Isoniazid inducible protein IniC	I6XV19	iniC	Rv0343	−7.74
Acyl-CoA ligase FadD31	I6Y7V6	fadD31	Rv1925	−7.97
Fatty-acid-CoA ligase FadD9	Q50631	fadD9	Rv2590	−7.15
Polyketide synthase Pks7	P94996	pks7	Rv1661	−4.42
Polyketide beta-ketoacyl synthase Pks4	I6Y9V4	pks4	Rv1181	−26.47
Conserved protein	O06216	Rv2161c	Rv2161c	−156.57
Methyltransferase	I6XFR4	Rv2952	Rv2952	−164.28
Methoxy mycolic acid synthase 3 MmaA3	I6XVV3	mmaA3	Rv0643c	−10.88
Phenolpthiocerol synthesis type-I polyketide synthase PpsC	I6X5S4	ppsC	Rv2933	36.43
Phenolpthiocerol synthesis type-I polyketide synthase PpsE	I6Y228	ppsE	Rv2935	6.30
Propionyl-CoA carboxylase beta chain 6 AccD6	I6XDV6	accD6	Rv2247	6.08
3-oxoacyl-[acyl-carrier-protein] synthase 1 KasA	I6Y8T4	kasA	Rv2245	5.18

## Discussion

This is the first study to generate information on the DA proteomic profile of *M. tuberculosis* lineage 7 vs. lineage 4 strains. We compared the proteomes in the two lineages and obtained 2867 protein groups that cover 71% of the predicted *M. tuberculosis* proteome (*n* = 4023). Former studies have documented even higher protein coverages with maximal ranges of 77–82% of the predicted *M. tuberculosis* proteome (Kelkar et al., 2011; Peters et al., 2016). Compared to recent publications that reported total protein coverages of 46% (Bespyatykh et al., 2016), 54% (Jhingan et al., 2016) and 62% (Schubert et al., 2015), the number of proteins identified in our study is relatively high.

This study showed considerable differences in the levels of protein abundance between *M. tuberculosis* lineage 7 and lineage 4 strains. Transporter proteins of the Pst system Rv0932 (PstS2), RV0933 (PstB), and RV0934 (PstS1) were less abundant in the two lineage 7 strains. PstA, PstC, PstS, and PstB form an ABC transporter essential for the capture of periplasmic inorganic phosphate (P_i_) in *M. tuberculosis* (Rifat et al., 2009). While PstS binds P_i_ with high affinity, PstB provides the energy required for P_i_ transport from the periplasm to the cytosol. A former study demonstrated that *M. tuberculosis* strains with a disruption in genes encoding the pst system were deficient in phosphate uptake and exhibited decreased virulence and attenuated growth, and this was attributed to the absence of the PstS2 protein (Peirs et al., 2005). In our study, PstS2 in lineage 7 strains was 261-fold lower than that in lineage 4. The phosphate starvation response (PSR) is an important mechanism for *M. tuberculosis* survival under phosphate-depleted conditions (Rifat et al., 2009). In this regard, the *M. tuberculosis* genes *regX3* and *phoR* are known to encode components that are essential for bacillary survival during phosphate limitation and for regulation of PSR (Rifat et al., 2009, 2014). In the two lineage 7 strains, we observed upregulation of the RegX3 and PhoR proteins that may suggest enhanced expression of PSR. P_i_ is an essential component of DNA, RNA, ATP, phospholipids, and proteins, and is crucial for energy transfer, protein activation, and carbon and amino acid metabolic processes (Tischler et al., 2013). The significant downregulation of essential proteins for P_i_ uptake in lineage 7 (L7-35 and L7-28) strains suggests that these proteins may contribute to the relatively slow *in vitro* growth described in our former study (Yimer et al., 2015).

This study showed downregulation of proteins involved in the type 7 secretion systems ESX-3 and ESX-1 in lineage 7 (L7-35 and L7-28) strains. ESX-1, ESX-3, and ESX-5 have been shown to be crucial for virulence or viability in *M. tuberculosis* (van Winden et al., 2016). The ESX-3 secretion machinery is encoded by an operon, including the genes *eccA3, eccB3, eccC3, eccD3, rv0291*, and *eccE3* (Table 1). All proteins encoded by these genes were downregulated in lineage 7 strains. The Esx-3 system is essential for siderophore-mediated iron acquisition, and is critical for *in vitro* growth of *M. tuberculosis* (Serafini et al., 2013). *M. tuberculosis* uses siderophore molecules (mycobactin and carboxymycobactin) to acquire iron. MmpS4/MmpL4 and MmpS5/MmpL5 proteins are essential for the biosynthesis and transport of siderophores in *M. tuberculosis* (Wells et al., 2013). In the current study, MmpL5 was downregulated in lineage 7 strains. A non-synonymous mutation in the *mmpL4* gene was identified in our recent WGS study (Yimer et al., 2016). The Rv0291 (MycP3) protein downregulated in lineage 7 strains is an essential protein for *M. tuberculosis* growth *in vitro* (Sassetti et al., 2003). The downregulation of several components of the essential type 7 secretion system ESX-3 may expose lineage 7 strains to iron starvation that potentially affects its growth *in vitro*.

A number of proteins that are involved in counteracting the effect of ROI and RNI were downregulated in the two lineage 7 strains compared to lineage 4 (Table 3). The protein CtpV is responsible for copper transport in *M. tuberculosis* and copper levels increase during hypoxia (White et al., 2009; Ward et al., 2010). Excess copper is toxic and must be regulated in *M. tuberculosis*. Several proteins, including CysD, CysN, CysH, and SirA involved in cysteine biosynthesis and sulfur metabolism, were downregulated in lineage 7. Cysteine is a precursor for glutathione biosynthesis (Kyoto Encyclopedia of Genes and Genomes; http://www.genome.jp/kegg/). The MshD and GgtB proteins are involved in mycothiol biosynthesis (Buchmeier et al., 2006) and glutathione metabolism (Kyoto Encyclopedia of Genes and Genomes; http://www.genome.jp/kegg/), respectively. Lineage 7 strains showed downregulation of ABC transporter proteins Rv1620c (CydC) and Rv1621c (CydD) that are involved in cytochrome biosynthesis (Kyoto Encyclopedia of Genes and Genomes; http://www.genome.jp/kegg/). CydDC contributes to nitric oxide tolerance (Holyoake et al., 2016). In contrast, several proteins that counteract the lethal effects of ROI and RNI in phagocytic cells were upregulated in the two lineage 7 strains (Table 3). GnD1 and ZwF1 proteins are required for glutathione metabolic process (Kyoto Encyclopedia of Genes and Genomes; http://www.genome.jp/kegg/). Rv3119 (MoaE1), Rv3324c (MoaC), and Rv3323c (MoaX) proteins are important in sulfur relay system (Kyoto Encyclopedia of Genes and Genomes; http://www.genome.jp/kegg/) and redox reaction in various compounds and are crucial for molybdenum cofactor (MoCo) biosynthesis (Williams et al., 2014). Molybdenum enzymes are involved in the sulfur, carbon, and nitrogen metabolism (Williams et al., 2014). In general, the downregulation of several proteins that counteract the effect of ROI and RNI highlights the exposure of lineage 7 cells to hostile environments. Conversely, the upregulation of specific proteins points to a compensatory mechanism adapted by lineage 7 strains to escape from the detrimental effect of ROI and RNI stress exposures.

Lineage 7 (L7-35 and L7-28) strains expressed various proteins involved in metabolic pathways that generate energy for survival. A number of proteins that play an important role in the TCA pathway were differentially expressed. While CitA, BkdB, and BkdA were downregulated, FrdA and LpdA proteins were upregulated in lineage 7 strains compared to lineage 4. The TCA is an aerobic core metabolic pathway which is crucial for the final steps of the oxidation of carbohydrates and fatty acids (Wikipedia^2^). The TCA pathway supplies NADH for use in oxidative phosphorylation and other metabolic processes. One study showed that in dormant cultivatable *M. tuberculosis*, the TCA was suppressed (Converse et al., 2009). When *M. tuberculosis* experiences hypoxia due to oxygen depletion, increased levels of NADH and inability to close the respiratory cycle, the bacteria shifts the direction of TCA enzymes from an oxidative direction to a reductive direction and starts to actively secrete succinic acid to complete the respiratory cycle. This process is facilitated by an enzyme called fumarate reductase (FrdA). We found an upregulation of FrdA that may signal a shift in metabolic pathway by lineage 7 strains. A former study showed that the FrdA protein was upregulated under hypoxic conditions in *M. tuberculosis* (Watanabe et al., 2011). Lineage 7 strains exhibited upregulation of LpdA protein; LpdA has quinone reductase activity and catalyzes the formation of NADH, which is important for energy production under anaerobic conditions (Zheng et al., 2008). As shown in Figure 6, FrdA and LpdA exhibit interactions with a number of proteins indicating the central role of these enzymes in energy metabolism in lineage 7 strains.

We also found upregulation of the CtaD, CtaC, QcrA, QcrC, and QcrB proteins that are components of the bc1-aa3 pathway. The bc1-aa3 cytochrome pathway is essential for growth *in vitro* and is the major respiratory route in mycobacteria (Matsoso et al., 2005). These proteins are also upregulated under hypoxia and poor-energy environments (Cook et al., 2014). In addition, the AceaA and AceaB enzymes involved in glyoxylate cycle that serves as an alternative to the TCA cycle, were upregulated in lineage 7. The GnD1 protein upregulated in lineage 7 strains functions in the pentose phosphate pathway as the main generator of cellular NADPH (Kyoto Encyclopedia of Genes and Genomes; http://www.genome.jp/kegg/). In addition, the PpgK protein involved in glucose phosphorylation (Kyoto Encyclopedia of Genes and Genomes; http://www.genome.jp/kegg/) was upregulated in lineage 7 strains.

*M. tuberculosis* is adapted to inhabit a wide range of intracellular and extracellular environments. A typical feature of this adaptation is the ability to respire and regenerate ATP via various energy-generating metabolic pathways (Cook et al., 2014). We observed downregulation of essential enzymes involved in core metabolic pathway of the TCA cycle. Therefore, the upregulation of the proteins specified above, which are involved in aerobic and anaerobic respiration in lineage 7 strains, may be considered as a compensatory mechanism for generating energy to maintain basic physiological functions *M. tuberculosis* lineage 7 cells.

Several proteins involved in cell wall/lipid biosynthesis were differentially expressed between the two lineage 7 strains and lineage 4. The Rv2952 protein, which is required for the biosynthesis of phenolglycolipid (PGL) and production of dimycocerosates of phthiocerol (DIM), was markedly downregulated in lineage 7 cells. Rv2952 is a methyltransferase that catalyzes the transfer of a methyl group for the production of DIM and PGL (Pérez et al., 2004). A study indicated that a Pks12 mutant strain was deficient in the synthesis of DIM, and that that the growth and virulence of this strain were reduced (Sirakova et al., 2003). Our recent study exhibited non-synonymous mutations in the *pkS12* gene (Yimer et al., 2016), suggesting a possible effect of this gene on the efficiency of PDIM synthesis. Similarly, the IniC protein downregulated in lineage 7 strains is suggested to participate in the regulation of cell wall growth (Alland et al., 2000). The downregulated MurE and MurF proteins are key enzymes of peptidoglycan biosynthetic pathway (Munshi et al., 2013). AccD6, KasA, PpsE, PpsC proteins were upregulated in lineage 7 strains compared to lineage 4. AccD6 and KasA proteins are key components in the mycolate biosynthesis (Bhatt et al., 2007; Pawelczyk et al., 2011). Mycolic acids are considered major virulence factors. PpsE and PpsC proteins are involved in phenolpthiocerol and phthiocerol dimycocerosate and important for virulence in *M. tuberculosis* (Bisson et al., 2012). The integrity of cell wall envelope is crucial for protection of *M. tuberculosis* against environmental stress factors. Therefore, the downregulation of a number of proteins involved in cell wall/lipid biosynthesis may have an effect on the growth and survival of *M. tuberculosis* lineage 7 cells.

This study addresses in detail the DA proteomic profile of *M. tuberculosis* lineage 7 (L7-35 and L7-28) vs. lineage 4 (H37Rv) strains. The analysis provides new insight into the lineage-specific protein profile variations that may explain the particular character of lineage 7 cells in terms of growth/survival and pathogenesis as compared to lineage 4. A number of proteins involved in *M. tuberculosis* growth and virulence fitness are less abundant in the two lineage 7 isolates in contrast to lineage 4. This may suggest that the *in vitro* slow-growth of *M. tuberculosis* lineage 7 bacilli and delayed health seeking among patients infected with lineage 7 strains observed in our earlier study may be phonotypic characteristics of lineage 7 cells. However, the most preferred method of linking phenotypic characteristics with a particular protein is to disrupt the gene encoding that protein and assess phenotypic alterations that the unavailability of that protein vests on the phenotype *in vitro* and in animal models. Therefore, further study by knocking out genes encoding the pst system and ESX-3 secretion system proteins, and observing phenotypic changes that the absence the corresponding proteins confers on the phenotype of lineage 7 vs. other lineages in *in vitro* and *in vivo* models, are warranted. In addition, further work examining the immune response of patients infected with lineage 7 vs. other lineages is imperative.

## Author contributions

TT and SY conceived the study and study design. SY collected the lineage 7 isolates. SY and EZ performed specimen handling and cultivation. AB, TR, and SK performed the MS analysis. SK, SY, and AB performed the bioinformatics analysis. SY, SK, AB, and TT evaluated and interpreted the data and drafted the paper. All authors edited and approved the final manuscript.

## Funding

Funding was received from the Research Council of Norway (RCN) FRIMEDBIO project 204747 and RCN GLOBVAC projects 234506 to TT and 192468 to CH, and Norwegian South-Eastern Health Authority project 2013080 to SY and TT.

### Conflict of interest statement

The authors declare that the research was conducted in the absence of any commercial or financial relationships that could be construed as a potential conflict of interest.

## Footnotes

^1^www.uniprot.org/help/gene_ontology

^2^https://en.wikipedia.org/wiki/Citric_acid_cycle
