# Supplementary figures and images for: Comparative Proteomic Analysis of Mycobacterium tuberculosis Lineage 7 and Lineage 4 Strains Reveals Differentially Abundant Proteins Linked to Slow Growth and Virulence
Source: Front Microbiol. 2017 May 9;8:795. doi: 10.3389/fmicb.2017.00795 (PMC5423352; doi:10.3389/fmicb.2017.00795)

**A***Mycobacterium tuberculosis* Lineages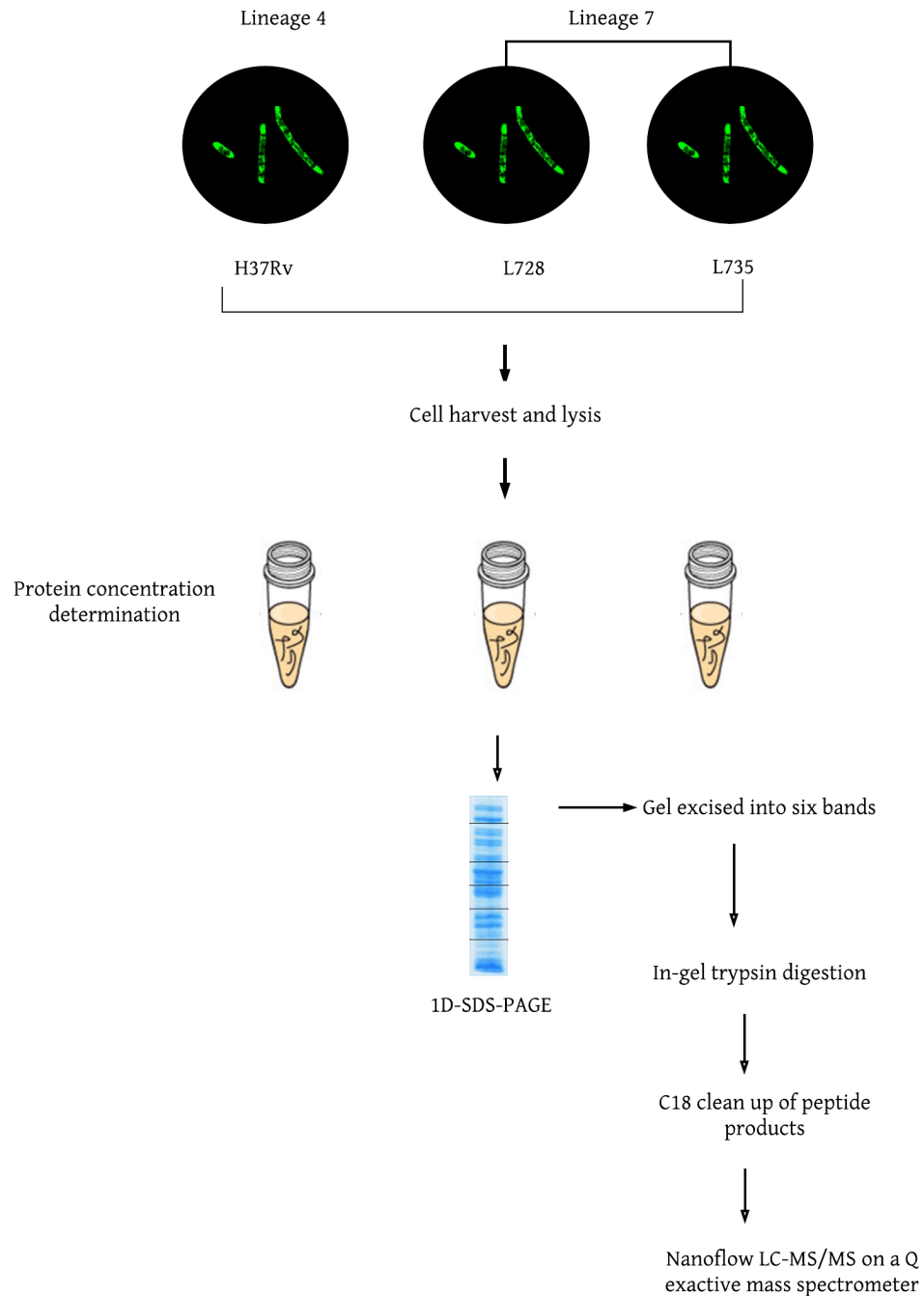**B**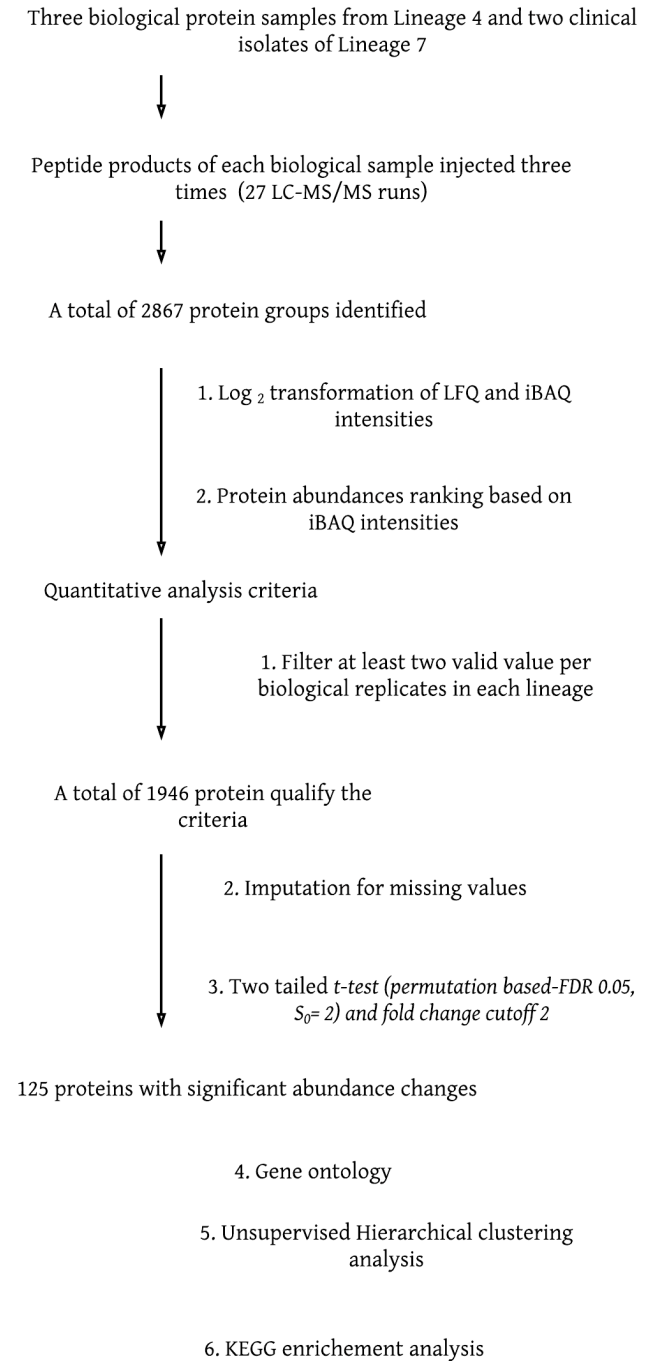

Supplement: Supplementary file 1 [file DataSheet1.pdf]
